# Enhancing of Women Functional Status with Metabolic Syndrome by Cardioprotective and Anti-Inflammatory Effects of Combined Aerobic and Resistance Training

**DOI:** 10.1371/journal.pone.0110160

**Published:** 2014-11-07

**Authors:** Ramires Alsamir Tibana, Dahan da Cunha Nascimento, Nuno Manuel Frade de Sousa, Vinicius Carolino de Souza, João Durigan, Amilton Vieira, Martim Bottaro, Otávio de Toledo Nóbrega, Jeeser Alves de Almeida, James Wilfred Navalta, Octavio Luiz Franco, Jonato Prestes

**Affiliations:** 1 Catholic University of Brasilia, Graduation Program on Physical Education, Brasilia, Brazil; 2 Laboratory of Exercise Physiology, Faculty Estacio of Vitoria, Espirito Santo, Brazil; 3 University of Brasilia, Brasilia, Brazil; 4 Department of Kinesiology and Nutrition Sciences of the University of Nevada, Las Vegas, Nevada, United States of America; 5 Centro de Analises Proteomicas e Bioquimicas. Programa de Pos-Graduaçao em Ciencias Genomicas e Biotecnologia, Universidade Catolica de Brasilia, Brasilia, Brazil; Indiana University Richard M. Fairbanks School of Public Health, United States of America

## Abstract

These data describe the effects of combined aerobic plus resistance training (CT) with regards to risk factors of metabolic syndrome (MetS), quality of life, functional capacity, and pro- and anti-inflammatory cytokines in women with MetS. In this context, thirteen women (35.4±6.2 yr) completed 10 weeks of CT consisting of three weekly sessions of ∼60 min aerobic training (treadmill at 65–70% of reserve heart rate, 30 min) and resistance training (3 sets of 8–12 repetitions maximum for main muscle groups). Dependent variables were maximum chest press strength; isometric hand-grip strength; 30 s chair stand test; six minute walk test; body mass; body mass index; body adiposity index; waist circumference; systolic (SBP), diastolic and mean blood pressure (MBP); blood glucose; HDL-C; triglycerides; interleukins (IL) 6, 10 and 12, osteoprotegerin (OPG) and serum nitric oxide metabolite (NOx); quality of life (SF-36) and Z-Score of MetS. There was an improvement in muscle strength on chest press (*p* = 0.009), isometric hand-grip strength (*p* = 0.03) and 30 s chair stand (*p* = 0.007). There was a decrease in SBP (p = 0.049), MBP (*p* = 0.041), Z-Score of MetS (*p* = 0.046), OPG (0.42±0.26 to 0.38±0.19 ng/mL, *p*<0.05) and NOx (13.3±2.3 µmol/L to 9.1±2.3 µmol/L; *p*<0.0005). IL-10 displayed an increase (13.6±7.5 to 17.2±12.3 pg/mL, *p*<0.05) after 10 weeks of training. Combined training also increased the perception of physical capacity (*p* = 0.011). This study endorses CT as an efficient tool to improve blood pressure, functional capacity, quality of life and reduce blood markers of inflammation, which has a clinical relevance in the prevention and treatment of MetS.

**Trial Registration:**

Brazilian Clinical Trials Registry (ReBec) - RBR-6gdyvz - http://www.ensaiosclinicos.gov.br/rg/?q=RBR-6gdyvz

## Introduction

Metabolic syndrome (MetS) combines several metabolic and hemodynamic alterations that increase the risks of cardiovascular diseases and premature death [1]. These risk factors include central obesity, dyslipidemia, hypertension and elevated fasting glucose [2]. Moreover, subjects with MetS present with 78% higher risk of cardiovascular events and death compared with healthy individuals [1]. According to the National Health and Nutrition Examination Survey (NHANES) conducted in the United States between 2003–2006, and the NCEP Adult Treatment Panel III (ATP III) guidelines, an estimated 34% of adults aged >20 years met the MetS criteria [3]. It has been shown that men between 20–50 years of age present higher MetS prevalence, while these numbers are higher in women after 50 years, which increases the attention of menopause transition period [4]. Additionally, the residual hypertension risk is increased by 90% in adult life, classifying this population as a target candidate for prevention from MetS risk factors [5].

Furthermore, MetS is also associated with chronic low-grade inflammation, a condition in which inflammatory blood markers are slightly elevated [6]. Interleukin-6 (IL-6), interleukin-1β (IL-1β) and tumor necrosis factor-α (TNF-α) cytokines play a central role on immune responses and inflammatory processes [7]. The activation of inflammation via Th1 lymphocytes through interleukin-12 (IL-12) has also shown to be involved [8]. Additionally, such inflammatory cytokines are known to contribute to the development of atherosclerosis, insulin resistance and hypertension, which are also criteria for MetS diagnosis [9], [10]. Another blood marker to be considered is osteoprotegerin (OPG), a soluble member of the TNF-α receptor superfamily produced by osteoblasts and vascular endothelial and smooth muscle cells [11], which may be involved in the regulation of vascular calcification and mortality [12].

Although nitric oxide (NO) has an important protective role in cardiovascular function, evidence indicates that higher NO levels are present in subjects with MetS [13] and diabetes [14]. Both disorders have been associated with increased atherogenic risk. Furthermore, elevated NO production may lead to insulin resistance [15] being another component to those of MetS, which may be involved in the disease pathophysiology [13].

Furthermore, it has been shown that increased levels of physical activity and functional capacity may reduce MetS incidence and seem to be key components for treatment and prevention [16]. Previous investigations have shown that chronic exercise training reduces the production of pro-inflammatory mediators, enhancing anti-inflammatory mediator activity, including interleukin-10 (IL-10). Such functional properties confer to exercise an important strategy to reduce the low grade inflammatory status of patients with MetS [17].

Studies with resistance training revealed improvements in lean mass, strength gain and reduced levels of inflammatory cytokines, while aerobic training is reported to be effective in improving maximal oxygen consumption (VO_2_max), high density lipoprotein (HDL) levels and reducing the level of triglycerides found in the plasma [18]-[20]. Moreover, the combination of aerobic and resistance training could optimize both cardiovascular and neuromuscular adaptations [21], which are extremely important for women with MetS.

According to Stensvold et al. [33], the optimal training regime to treat MetS and its associated cardiovascular abnormalities remains uncertain. Furthermore, no previous study focused on the effects of combined training on OPG, NO and quality of life in women with MetS.

Thus, this study aims to evaluate the effects of combined aerobic and resistance training (CT) on cardiometabolic risk factors, inflammatory blood markers, quality of life and functional capacity in adult women with MetS. The initial hypothesis is that CT improves cardiometabolic profile, increases functional capacity and reduces inflammatory blood markers in women with MetS.

## Materials and Methods

### 

#### Ethics Statement

The protocol for this trial and supporting CONSORT checklist are available as supporting information; see Checklist S1 and Protocol S1.

All participants provided and signed a written informed consent document prior to participation in the study. All procedures were approved by the Catholic University of Brasilia Research Ethics Committee for Human Use at November, 29, 2012 (protocol CEP/UCB, #279/2010) and the study was conducted according to the principles expressed in the Declaration of Helsinki. The study was conducted at the laboratory of resistance training of the Catholic University of Brasilia.

The trial of this study was registered after patient recruitment began, because only the approval of the Research Ethics Committee was necessary to conduct the study at the Catholic University of Brasilia. The authors confirm that all ongoing and related trial for this intervention is registered.

#### Participants

Initially, 20 women from the local community (Vila Telebrasilia, Brazil) volunteered to participate and were recruited from posters and lectures about the study from January to April. Each participant received personal explanation after being informed about the study protocol. Although verbal encouragement was given to increase compliance during follow-up (May to July), only 13 completed the study, with two excluded due to caloric restriction and five excluded from the statistical analysis because they missed more than 25% of the training sessions. Individuals completed a thorough physical examination, including a medical history, resting and exercise electrocardiogram [22]. Moreover blood pressure assessments, anthropometric and orthopedic evaluations prior to participation in the experimental protocols were also performed. As inclusion criteria, the only participants included were those aged between 18–40 y, classified with MetS, and those without consistent resistance training for the past six months before the study period. According to ATP III [2], MetS is defined as the presence of 3 or more of the following 5 criteria: increased waist circumference (≥88 cm), hypertriglyceridemia (≥150 mg/dL), low HDL (<50 mg/dL), hypertension (≥130/85 mm Hg), and high fasting glucose (≥110 mg/dL). A modified z score was calculated for each variable using individual subject data using the ATP III criteria. The equations used to calculate the MetS z score were as follows: {z score  =  [(50 - HDL)/11.8] + [(TG −150)/66.2] + [(fasting blood glucose - 110)/10.4] + [(waist circumference - 88)/9.2] + [(mean arterial pressure - 100)/8.7]/100}. Women with physical disabilities, under caloric restriction, diagnosis of diabetes, cardiovascular diseases, uncontrolled hypertension (systolic blood pressure >140 mmHg and diastolic blood pressure >90 mmHg), musculoskeletal disease, recent use of medication and smoking or drug/alcohol abuse were excluded from the trial. A sedentary state was defined by the International physical activity questionnaire (IPAQ).

#### Study design

We conducted a 10-week prospective quasi-experimental design. Participants and those assessing the outcomes were not blinded to study condition assignment. Subjects were advised to maintain their normal daily eating habits throughout the study. Prior to physical evaluation, participants reported to the laboratory between 08:00–10:00 am following an overnight fast, for blood sampling from the antecubital vein for subsequent analysis of the biochemical variables. Volunteers completed two weeks of familiarization prior to testing (3 sessions/week, with one exercise for each main muscle group which were the same exercises used during resistance training), where they were advised regarding the execution of proper technique. After the familiarization period, one-repetition maximum (1-RM) test and re-test were performed on the chest press on two nonconsecutive days with 48–72 h between tests. The CT protocol began three days after 1-RM testing and was performed on three non-consecutive days of the week, comprising 30 min of moderate to moderate-high intensity on a treadmill at 70–80% reserve heart rate and three sets of 8–12 repetition maximum (RM) of twelve exercises for whole body, and 1-minute rest intervals between sets and exercises.

#### Exercise Training

Training sessions lasted ∼60 min in a facility dedicated to research and under the supervision of exercise physiologists.

#### Aerobic training

The aerobic component of the program consisted of 30 min of moderate to moderate-high intensity on a treadmill at 70–80% reserve heart rate, with heart rate being continuously monitored throughout each session.

#### Resistance training

Subjects completed two weeks of familiarization prior to the resistance training program. During the familiarization weeks, individuals were advised regarding proper resistance training technique and completed 3 sessions/week, with one exercise of each main muscle group consisting of 3 sets of 10–12 submaximal repetitions. After the familiarization period subjects initiated the resistance training program consisting of 3 sessions/week during ten weeks. Resistance training machines were from JOHNSON (Landmark Drive, Cottage Grove, USA). All training sessions were carefully supervised by three experienced professionals (ratio of supervision 1∶2 – 1 professor for 2 participants). Participants were required to complete at least 85% of the exercise sessions. No major complications or cardiac events occurred during the study period. The resistance training was divided into A (leg press, knee extensor, leg curl, hip adduction, hip abduction, standing calf raise and abdominal crunches), B (chest press, lat pull down, shoulder press, machine elbow flexion, triceps extension and abdominal crunches) and C (leg press, knee extensor, leg curl, chest press, lat pull down, shoulder press and abdominal crunches) regiments. For all listed exercises, three sets with 8–12 RM were performed, with a one-minute rest interval between each set and exercise. Training loads were monitored each session according to the increase in muscle capacity of the participants. The mean duration to complete one repetition was 3–4 s (both concentric and eccentric phases of the movement) and training sessions lasted ∼30 min. The number of repetitions and the loads used for each exercise session were recorded. The loads were updated when necessary to keep the number of repetitions within the same range of RM and to provide a progressive overload. Additionally, correct breathing patterns were instructed to avoid Valsalva maneuver.

## Outcome Measures

### Primary Outcomes

#### Cytokines

Participants reported to the laboratory between 08:00–10:00 am, after an overnight fast, and after blood collection, samples were centrifuged at room temperature at 2,000 rpm for 15 min. All subjects were encouraged to avoid smoking, alcohol and caffeine consumption as well as unusual physical activity to avoid influence on these parameters. The serum was removed and frozen at 80°C for further analysis. Serum was analyzed for amyloid A using a DADE Dimension RXL clinical chemistry analyzer (Dade-Behring, Inc, Newark, DE, USA). The analyzer was calibrated daily using Liquid-Assayed Multiqual (Bio-Rad, Hercules, CA, USA), and two levels of quality control with known concentrations. In addition, serum IL-10, IL-6 and IL-12 were assessed using commercially available enzyme-linked immunosorbent assay (ELISA) kits (BioLegend's ELISA Max Deluxe, San Diego, CA, USA) and OPG (R&D System Inc., Minneapolis, MN, USA). Standard curves were generated using commercially available microplate reader-compatible statistical software (MicroWin 2000, Microtek Laborsysteme GmbH, Overath, Germany). All samples were determined in duplicate to guarantee the precision of the results. For all measures the mean intra-assay coefficient of variation was 2.9–9.5%, the inter-assay coefficient of variation was 5.9–7.0%, and the sensitivity was 0.0093 pg/mL.

#### Nitrite Levels

To perform the analysis, the blood was centrifuged previously. The serum concentration of nitrite was determined by the Griess reaction. In brief, serum samples were deproteinized by adding zinc sulfate (15 mg/mL), followed by centrifugation at 10000 g for 10 min; 100 µL of the supernatant was applied to a microplate well, and following addition of 100 µL vanadium (III) chloride (8 mg/mL) to each well, Griess reagents [50 µL sulfanilamide (2%) and 50 µL N- (1-naphthyl) ethylendiamine dihydrochloride (0.1%)] were added. After 30 min incubation at 37°C, absorbance was read at 540 nm using the ELISA reader (Sunrise, Tecan, Austria). Concentration of NO_2_ in serum samples was determined from the linear standard curve established by 0–100 µM sodium nitrite.

### Secondary Outcomes

#### Maximal strength testing

After 2 weeks of adaptation to the exercises and 3–5 days after the last training session, 1-RM test was performed on chest press exercise on 2 different days separated by a minimum of 48 h. The protocol consisted of a light warm-up of 5 min of treadmill walking followed by 8 repetitions at 50% of estimated 1-RM (according to the participants' capacity verified in the 2 weeks of adaptation). After a 1-min rest, subjects performed 3 repetitions at 70% of the estimated 1-RM. Following 3 minutes of rest, participants completed 3–5 attempts interspersed with 3-to-5 min rest intervals, with progressively heavier weights (∼5%) until the 1-RM was determined.

#### Isometric handgrip strength

Isometric handgrip strength was determined by a manual mechanical dynamometer (Takei, T.K.K Grip strength dynamometer 0–100 kg, Japan). Volunteers stood still with both arms extended and the forearm positioned in a neutral rotation. The handgrip width of the dynamometer was individually adjusted for each participant, according to hand size allowing the stem next to the body to be positioned on the second phalanges: index, medial and ring fingers. Three attempts were allowed interspersed with 1 min rest intervals. The best measure was used and relative isometric strength was determined as follows: Relative strength  =  Absolute strength (kg)/Body mass (kg) [23].

#### The 6-minute walk test

The 6-minute walk test (6MWT) was performed according to the American Thoracic Society protocol [24]. Functional capacity was determined by the distance walked in a covered 30-meter walkway.

#### Functional fitness tests

Two components of the functional fitness test [25] were selected as a measure of functional performance. The following test items were conducted as follows: (1) a 30-s chair stand test (the maximum number of times within 30 s that an individual can rise to a full stand from a seated position, without pushing off with the arms), and (2) the sit and reach test evaluated flexibility using the modified chair sit-and-reach test.

#### Anthropometrics

Height and weight were measured for the calculation of the body mass index (BMI). All circumferences were obtained using non elastic tape, and measurements were obtained in triplicate and averaged to obtain the circumference score. Neck circumference was obtained with the subject sitting with the head in the Frankfort horizontal plane position. Briefly, a measuring tape was applied around the neck inferior to the laryngeal prominence and perpendicular to the long axis of the neck, while the minimal circumference was measured and recorded to the nearest 0.1 cm [26]. Waist circumference was measured at the midpoint between the lower rib margin, hip circumference was measured at the widest portion of the buttocks and the body adiposity index (BAI) was determined by the following formula: (BAI  =  [(hip circumference)/((height)1.5)–18)] [27].

#### Blood pressure measurement

Systolic (SBP), diastolic (DBP) were measured with a validated oscillometric device (Microlife 3AC1-1, Widnau, Switzerland) according to the recommendations of the International Protocol of the European Society of Hypertension [28]. The cuff size was adapted to the arm circumference of each participant according to the manufacturer's recommendations, and BP was assessed in triplicate (measurements separated by 5 min) with the mean value used for further analysis after 10 min of seated rest. All BP measurements were taken on the left arm. Mean blood pressure (MBP) was calculated as the sum of DBP and one-third of the pulse pressure. All blood pressure measures were assessed in triplicate (measurements separated by 1 min) with the mean value used for analysis.

#### Biochemical parameters

Participants reported to the laboratory between 08:00–10:00 am, after an overnight fast, for blood withdrawal from the antecubital vein. Plasma triglycerides, HDL-cholesterol and glucose levels were measured by enzymatic CHOP-POD, homogeneous HDL-cholesterol and Hexokinase methods, respectively.

#### Quality of life

Health-related quality of life was assessed using the Brazilian version of the Short Form 36 Health Survey (SF-36) [29]. Quality of life is a multidimensional construction characterized by four essential components: psychological well-being; physical functioning; social relations; and functional competence. The measure assesses eight dimensions. For each item, patients were required to select the answer closest to their own experience. The types of answer possible varied from item to item, from yes or no answers to answers on a scale of 1–6. Each domain score ranges from 0 to 100, the highest score indicating better health conditions related to quality of life.

### Sample size

The power of the sample size was determined using G*Power version 3.1.3, based on the differences between pre and post primary outcomes and the metabolic syndrome z score. Considering the final sample size of this study and an alpha error of 0.05, the sample power (1−β) was 1.00 for NO, 0.96 for IL-10/NO ratio and 0.71 for metabolic syndrome z score. Unfortunately, IL-6 (1−β = 0,24), IL-12 (1−β = 0,25), IL-10 (1–β = 0,60) and OPG (1–β = 0,57) did not reach the recommended power and interpretation of the data must be careful, specially the IL-6 and the IL-12. However, the effect sizes of the correlations involving IL-6 and IL-12 with anthropometric or biochemical variables in this study were large (|*p*|>0.5), corresponding to a strong sample power for the correlations [30].

### Statistical analysis

The results are expressed as means ± standard deviation (SD). Shapiro-Wilk tests were applied to check for normality distribution of study variables. In case of nonparametric distribution, a logarithmic transformation was performed. The anthropometric, biochemical, NO and physical fitness variables were parametrically distributed, however, the SF-36 scores, cytokines and osteoprotegerin were nonparametriclly distributed. The pre and post-intervention variables were compared using paired-samples t-test (parametric samples) and the Wilcoxon signed-rank test (nonparametric samples). The Spearman's correlation was used to evaluate the correlation between delta (post-pre) cytokines concentration and anthropometric or biochemical variables and between physical fitness and SF-36 scores after training program. The Cohen's convention (1988) [30] was used for assigning strength of association between the variables. The level of significance was p≤0.05 and SPSS version 20.0 (Somers, NY, USA) software was used.

## Results

### 

#### Flow of participants through trial

**Figure 1:**
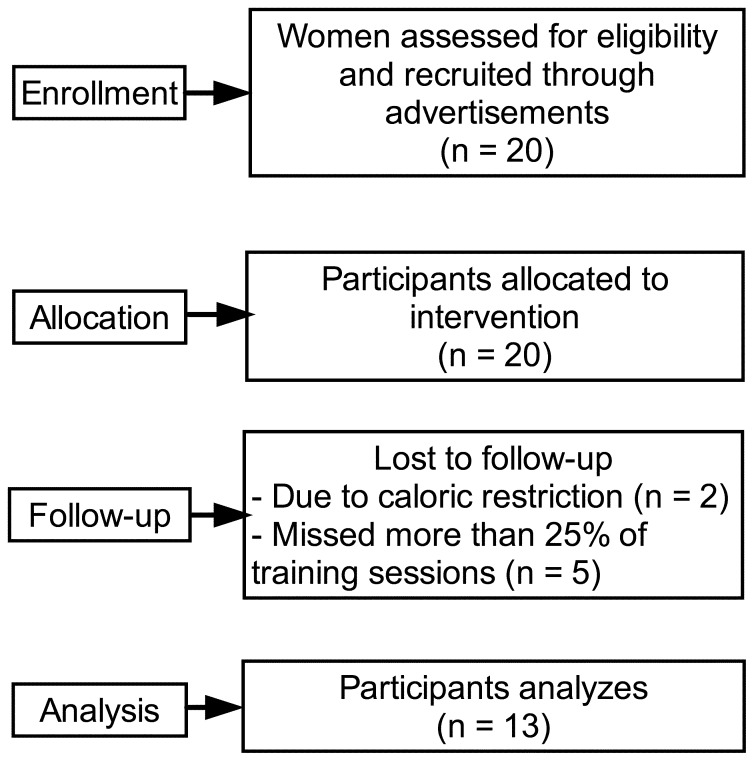
Flow-chart of the participants.

Initially, 20 women volunteered to participate. Only 13 completed the study, with two excluded due to caloric restriction and five excluded from the statistical analysis because they missed more than 25% of the training sessions (figure 1).

#### Subject Characteristics

On average, volunteers attended 20±3 training sessions during the 10-week training program. Table 1 presents the anthropometric and hemodynamic characteristics of the subjects before and after the training program. There were no statistically significant differences after 10-weeks of the training program with respect to anthropometric variables (*p*≥0.05).

**Table 1:** Anthropometric and hemodynamic characteristics of the subjects (n = 13) before and after the training program (mean ± SD).

	Pre	Post	*p*-value
Weight, kg	74.8±7.2	74.2±7.5	0.352
Body mass index, kg/m^2^	30.0±2.5	29.7±2.0	0.321
Waist circumference, cm	85.8±4.9	84.9±5.6	0.237
Hip circumference, cm	105.9±4.5	105.1±4.3	0.242
Neck circumference, cm	35.2±2.1	35.7±1.6	0.165
Waist to height ratio	0.54±0.04	0.54±0.03	0.468
Waist to hip ratio	0.81±0.04	0.81±0.05	0.514
Body adiposity index, %	26.8±2.6	26.4±1.9	0.238
Systolic blood pressure, mmHg	119.2±14.6	112.7±9.7	0.049*
Diastolic blood pressure, mmHg	79.5±15.6	72.8±10.1	0.051
Mean arterial pressure, mmHg	92.8±14.6	86.1±8.8	0.041*
Metabolic syndrome z score	1.26±0.17	1.19±0.09	0.046*

*Statistically significant (p≤0.05).

#### Metabolic Risk Factors

Analyses of HDL (44.5±9.5 mg/dL pre to 46.4±8.8 mg/dL post training, *p* = 0.392), glucose (88.6±8.4 mg/dL pre to 88.3±4.6 mg/dL post training, *p* = 0.882) and triglyceride (118.0±56.5 mg/dL pre to 118.5±61.8 mg/dL post training, *p* = 0.910) concentrations did not reveal any significant changes. However, there was a significant decrease in systolic (p = 0.049), mean arterial pressure (p = 0.041) and metabolic syndrome z score (p = 0.046).

#### Inflammation blood markers

**Figure 2:**
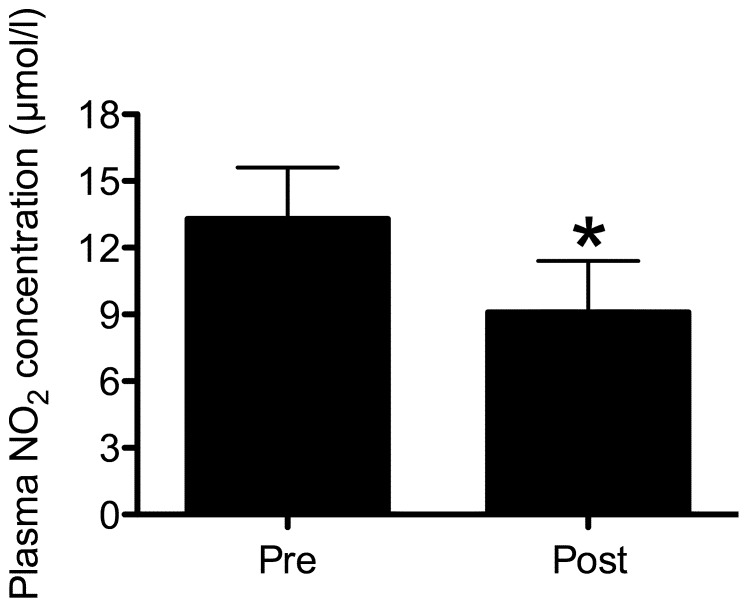
Nitric oxide (NO) concentration before (pre) and after (post) the training program (mean ± SD). *Statistically different compared with pre-values (p≤0.05).

After the 10-week training program, the NO plasma concentration had a significant decrease (13.3±2.3 µmol/L to 9.1±2.3 µmol/L; p<0.0005; figure 2). Interestingly, NO was reduced after the training program for all volunteers. The minimum decrease was approximately 15% and the maximum was 60% (31.8±12.1%). There was a significant increase in IL-10 concentration (*p* = 0.043) and a significant decrease in OPG concentration (*p* = 0.034) after the training program. There were no statistically significant differences (*p*≥0.05) pertaining to IL-6 or IL-12 concentrations after the training program (table 2). The ratio IL-10/NO also displayed a significant increase (*p* = 0.008) as a result of the training program. The Spearman's correlations between cytokine concentration and anthropometric or biochemical variables are presented in table 3. According to Cohen (1988), the strength of association for all significant correlations (p≤0.05) presented in this study were large correlations.

**Table 2:** Evaluation of Interleukin-10 (IL-10), osteoprotegerin (OPG), interleukin-6 (IL-6) and interleukin-12 (IL-12) concentrations before and after the training program (mean ± SD).

	Pre	Post	*p*-value
IL-10, pg/mL	14.96±7.48	19.38±12.30	0.043*
OPG, pg/mL	0.49±0.26	0.43±0.19	0.034*
IL-6, pg/mL	0.63±1.95	0.13±0.33	0.276
IL-12, mg/dL	1.04±1.49	0.66±0.89	0.237
IL-10/NO	1.21±0.53	2.35±1.36	0.008*

*Statistically significant (p≤0.05).

**Table 3:** Spearman's correlations between cytokines concentration and anthropometric or biochemical variables.

	IL-10	OPG	IL-6	IL-12
**Delta (post-pre) correlations**				
Neck circumference	NS	NS	0.616*	NS
Body adiposity index	NS	NS	0.637*	0.572*
HDL	NS	NS	0.649*	0.723*
NO	NS	NS	NS	NS

IL-10, interleukin-10; OPG, osteoprotegerin; IL-6, interleukin-6; IL-12, interleukin-12; HDL, high-density lipoprotein; NO, nitric oxide; NS, non-significant; *Statistically significant (p≤0.05).

#### Functional fitness tests

Table 4 presents the physical fitness tests performed before and after the training program. There was a significant increase for the sitting-rising test (*p* = 0.007), and chest and handgrip strength (*p*≤0.05) after completion of the training program. There were no statistically significant increases in flexibility (*p* = 0.402) or the 6-min walking tests (*p* = 0.606).

**Table 4:** Physical fitness tests performed before and after the training program (mean ± SD).

	Pre	Post	*p*-value
Sitting-rising, rep	20.3±4.0	24.5±4.6	0.007*
Flexibility, cm	28.6±9.3	29.3±8.7	0.402
6-min walking, m	587.4±42.9	581.3±48.2	0.606
Chest press 1RM, kg	55.8±6.8	59.8±9.4	0.009*
Chest press relative strength	0.75±0.09	0.81±0.10	0.002*
Right handgrip, kg	33.7±7.7	35.0±7.7	0.030*
Right handgrip relative strength	0.45±0.09	0.47±0.08	0.008*
Left handgrip, kg	31.1±7.2	32.5±6.1	0.024*
Left handgrip relative strength	0.41±0.09	0.44±0.07	0.016*

1RM, 1-repetition maximum.

*Statistically significant (p≤0.05).

#### Quality of life

The SF-36 scores were shown in table 5. Among the eight domains of the SF-36, physical function scale was the only one that had a significant increase (*p* = 0.011) after the training program. There were positive correlations of post 6-min walking test score with post SF-36 scores of physical function (*r* = 0.586, *p* = 0.035), role physical (*r* = 0.582, *p* = 0.037), general health (*r* = 0.631, *p* = 0.021) and role emotional (*r* = 0.676, *p* = 0.011). The post flexibility score after the training program also had a positive correlation with the post SF-36 score of general health (*r* = 0.645, *p* = 0.017).

**Table 5:** SF-36 scores before and after the training program (mean ± SD).

	Pre	Post	*p*-value
Physical function	76.9±21.0	82.7±19.5	0.011*
Role Physical	75.0±28.9	76.9±37.4	0.666
Bodily pain	60.3±28.1	65.9±23.0	0.498
General health	56.1±17.6	59.0±14.8	0.674
Vitality	57.7±10.3	57.7±12.0	0.300
Social function	78.9±21.9	81.7±23.7	0.944
Role emotional	74.3±30.9	69.2±39.6	0.683
Mental health	58.8±7.7	58.4±11.2	0.860

*Statistically significant (p≤0.05).

## Discussion

### Interpretation

The main findings of the present study provide evidence that 10 weeks of CT decrease chronic inflammation, blood pressure, Z-Score of MetS, and improve muscle strength, quality of life and functional capacity in adult women with MetS, confirming the initial hypothesis. Moreover, although there was no weight loss, women with MetS decreased blood markers of low-grade inflammation associated with overweight and obesity.

CT is widely used due to the unique advantages of aerobic and resistance training. Aerobic training has been shown to improve cardiorespiratory fitness, promoting energy expenditure and fat utilization, while resistance training induces gains in muscle size, strength, endurance, and power. Furthermore, CT has been reported not only to reduce health risks and symptoms associated with physical inactivity, but improves performance of daily living activities [21], [31].

MetS is associated with chronic low-grade inflammation, a condition in which inflammatory markers in the circulation are slightly elevated [32]. In our study, there was a significant decrease in blood markers of inflammation (OPG and NO) and an increase of anti-inflammatory cytokines (IL-10) following ten weeks of CT. The change in IL-6 and IL-12 could not be shown, however, because the lack of sample power for these variables, the result warrants confirmation. Meanwhile, it was shown a large correlation between the change in body adiposity index and IL-6 or IL-12 concentrations after CT. Recently, Stensvold et al (2012) [33] reported that after 12 weeks of aerobic interval training the levels of TNF-α were lower as compared to resistance trained and control groups in subjects with MetS. However, different from the present study, total body fat was reduced with aerobic interval training (from 33.9±7.3% to 32.2±7.9%) and resistance training (from 31.2±3.9% to 29.7±3.4%). Similarly, Ho et al (2013) [34] found that 12 weeks of moderate-intensity aerobic (60% heart rate reserve), resistance training (four sets of 8–12 repetitions at 10-RM level of 5 whole body exercises), and CT (15 min of aerobic exercise and 2 sets of 8–12 repetitions of 5 whole body exercises) decreased TNF-α in overweight and obese individuals compared to a control group.

Growing evidence suggests that NO plays an important role in the development of MetS [35], [13]. Previous studies reported that increased plasma NO concentration in hypertensive coronary artery disease patients might represent a compensatory response to increased superoxide anion concentration [36]. Moreover, increased shear stress with hypertension might stimulate NO production. Zahedi et al (2008) [13] also have found similar results and suggested that nonspecific tissue damage in MetS and diabetes might increase the activity of inducible nitric oxide synthase (iNOS), and therefore results in the accumulation of NO. In our study, there was a significant decrease in NO levels following 10 weeks of CT. The decrease in NO and OPG in response to CT reveal important protective effects against the deleterious effects of low-grade inflammation in women with MetS.

The increase in the anti-inflammatory response following CT, represented by the increased levels of IL-10 may have contributed to the decrease of blood markers of inflammation (OPG and NO), as shown in previous studies [37], [38].

Nevertheless, data reveal that despite improvements in the cardiovascular risk factors of MetS (blood pressure and inflammation) and the increase in functional capacity, no modifications with respect to blood glucose, HDL-C and triglycerides were observed. Similarly, meta-analytic data revealed that isolated exercise programs without caloric restriction induced limited improvements in such parameters. Shaw et al (2006) [39] evaluated 43 studies including 3476 participants and found that exercise without caloric restriction control is associated with a lower decrease of body mass, blood pressure and blood glucose as compared with exercise associated with dietary restriction. Orozco et al (2008) [40] compared the effects of isolated diet and diet plus CT. Results revealed that diabetes risk was lower in the combined group. Additionally, individuals submitted to combined exercise training and diet presented a decrease in blood pressure and obesity anthropometric indexes, which was not observed for the group participating in exercise alone.

Regarding the quality of life, it has been reported that subjects with MetS have reduced perception of quality of life [40]. Recently, Okosun et al (2013) [41] found that MetS is associated with poor physical health (1.65; 95% CI = 1.09−2.50), mental health (1.67; 95% CI = 1.09−2.83) and overall health (1.44; 95% CI = 0.80−2.59) in Mexican-Americans. In the present study, it was observed that CT improved the perception of physical function in adult women with MetS. Our findings were similar to previous trials in subjects with type 2 diabetes [42] and obesity [43]. Imayama et al (2011) [44] examined the individual and combined effects of dietary weight loss and/or exercise interventions (moderate to vigorous aerobic exercise/225 min per week) on health-related quality of life (HRQOL) in overweight/obese postmenopausal women. This same report showed that a combined diet and exercise intervention had positive effects on HRQOL and psychological health, which may be greater than that of exercise or diet alone. Thus, it is likely that the CT program utilized in the present study would provide additional improvements in quality of life parameters if combined with a dietary restriction intervention.

The present study has some limitations that should be considered, such as the limited time of the intervention (only ten weeks), the reduced sample power for cytokines, and the lack of more accurate measures to evaluate body composition (body fat and lean body mass), which may have influenced the results. Another limitation was the lack of a control group, although it has been speculated that a control group is not always necessary, particularly considering that the health of the subjects could be compromised as a result of not participating in CT. Additionally, results from a control group would probably reveal no positive effect on anthropometric, biochemical and cardiovascular risk factors of MetS, as has been reported in a previous study [44]. Finally, the non-significant variation with the biochemical data, such as glucose, triglycerides and HDL-C can be, at least in part attributed to the lack of dietetic control, as well as the limited sample size. It is possible that an interdisciplinary approach would induce more positive results in biochemical and anthropometric variables, as previously found in other studies. However, “significant” results based upon this exploratory research should clearly be labeled as exploratory results. To confirm these results the corresponding hypotheses have to be tested in further confirmatory studies.

In summary, ten weeks of CT reduced blood markers of inflammation such as NO, IL-10 and OPG, blood pressure and metabolic syndrome Z scores in women with MetS. Moreover, the improvement of the quality of life (physical function domain) and functional capacity (leg, chest and handgrip strength) reinforce the clinical relevance of CT for women with MetS. Thus, the results of this study provide further evidence for the recommendation of regular CT to decrease cardiovascular risk factors of MetS.

## Supporting Information

Checklist S1 **Supporting CONSORT checklist.**
(PDF)

Protocol S1 **Copy of the original protocol.**
(JPG)
