# Supplementary material for: Enhancing of Women Functional Status with Metabolic Syndrome by Cardioprotective and Anti-Inflammatory Effects of Combined Aerobic and Resistance Training
Source: PLoS One. 2014 Nov 7;9(11):e110160. doi: 10.1371/journal.pone.0110160 (PMC4224372; doi:10.1371/journal.pone.0110160)
Supplement: Checklist S1 — Supporting CONSORT checklist. (PDF) [file pone.0110160.s001.pdf]

Paper Section/ Topic	Item Number	Descriptor	Reported	
			**V**	**Pg#**
		**Title and Abstract**		
Title and Abstract	1	Information on how Unit were allocated to intervention	V	2 - Abstract
		Structured abstract recommended	V	2 - Abstract
		Information on target population or study sample	V	2 - Abstract
		Introduction		
		Scientific background and explanation of rationale	V	3 - Introduction
Background	2	Theories used in designing behavioral intervention	V	4 - Introduction
		**Methods**		
Particpants	3	Eligibility criteria for participants, including criteria at different levels in recruitment/sampling plan (e.g., cities, clinics, subjects)	V	5 - Participants
		Method of recruitment (e.g., referral, self-selection), including the sampling method if a systematic sampling plan was implemented	V	5 - Participants
		Recruitment setting	V	5 - Participants
		Settings and locations where the data were collected	V	5 - Participants
		* Details of the interventions intended for each study condition and how and when they were actually administered, specifically including		
		Content: what was given?	V	7 - Study design
		Delivery method: how was the content given?	V	7 - Study design
		Unit of delivery: how were the subjects grouped during delivery?	V	7 - Study design
		Deliverer: who delivered the invertion?	V	7 - Study design
		Exposure quantity and duration: how many session or epidoses or event were intended to be delivered? How long were they intended to last?	V	7 - Study design
		Time span: how long was it intended to take to deliver the intervention to each unit?	V	7 - Study design
Intervention	4	Activities to increase compliance or adherence (e.g., incentives)	V	7 - Study design
Objectives	5	Specific objectives and hypotheses	V	4 - Introduction
		Clearly defined primary and secondary outcome measures	V	7 - Study design
		Methods used to collect data and any methods used to enhance the quality of measurements	V	7 to 11 - Study design to outcome measures
Outcomes	6	Information on validated instruments such as psychometric and biometric properties	V	7 to 11 - Study design to outcome measures
Sample Size	7	How sample size was determined and, when applicable, explanation of any interim analyses and stopping rules'	V	12 - Sample
		Unit of assignment (the unit being assigned to study condition, e.g., individual, group, community)	V	5 - Participants
		Method used to assign units to study conditions, including details of any restriction (e.g., blocking, stratification, minimization)	V	5 - Participants
Assignement Method	8	Inclusion of aspects employed to help minimize potential bias induced due to non-randomization (e.g., matching)	V	5 - Participants
Blinding (masking)	9	Whether or not participants, those administering the interventions, and those assessing the outcomes were blinded to study condition assignment; if so, statement regarding how the blinding was accomplished and how it was assessed.	V	6 - Study design
		Description of the smallest unit that is being analyzed to assess intervention effects (e.g., individual, group, or community)	V	6 - Study design
Unit of analysis	10	If the unit of analysis differs from the unit of assignment, the analytical method used to account for this (e.g., adjusting the standard error estimates by the design effect or using multilevel analysis)	V	-
		Statistical methods used to compare study groups for primary methods outcome(s), including complex methods of correlated data	V	13 - Statistical Analysis
		Statistical methods used for additional analyses, such as a subgroup analyses and adjusted analysis	V	13 - Statistical Analysis
		Methods for imputing missing data, if used	V	13 - Statistical Analysis
Statistical Methods	11	Statistical software or programs used'	V	13 - Statistical Analysis
		**Results**		
		*Flow of participants through each stage of the study: enrollment, assignment, allocation, and intervention exposure, follow-up, analysis (a diagram is strongly recommended)	V	13 - Results
		Enrollment: the numbers of participants screened for eligibility, found to be eligible or not eligible, declined to be enrolled, and enrolled in the study	V	13 - Results
		Assignment: the numbers of participants assigned to a study condition	V	13 - Results
		Allocation and intervention exposure: the number of participants assigned to each study condition and the number of participants who received each intervention	V	13 - Results
		Follow-up: the number of participants who completed the follow-up or did not complete the follow-up (i.e., lost to follow-up), by study condition	V	13 - Results
		Analysis: the number of participants included in or excluded from the main analysis, by study condition	V	13 - Results
Participant flow	12	Description of protocol deviations from study as planned, along with reasons	V	13 - Results
		Dates defining the periods of recruitment and follow-up	V	5 - Participants
		Baseline demographic and clinical characteristics of participants in each study condition	V	5 - Participants
		Baseline characteristics for each study condition relevant to specific disease prevention research	V	5 - Participants
		Baseline comparisons of those lost to follow-up and those retained, overall and by study condition	V	5 - Participants
Recruitment	13	Comparison between study population at baseline and target population of interest	V	5 - Participants
		Baseline demographic and clinical characteristics of participants in each study condition	V	25-30 - Tables and Figures
		Baseline characteristics for each study condition relevant to specific disease prevention research	V	25-30 - Tables and Figures
		Baseline comparisons of those lost to follow-up and those retained, overall and by study condition	V	25-30 - Tables and Figures
Baseline data	14	Comparison between study population at baseline and target population of interest	V	25-30 - Tables and Figures
Baseline equivalence	15	Data on study group equivalence at baseline and statistical methods used to control for baseline differences	V	25-30 - Tables and Figures
		Number of participants (denominator) included in each analysis for each study condition, particularly when the denominators change for different outcomes; statement of the results in absolute numbers when feasible	V	12 to 13 - Sample Size
Numbers analyzed	16	Indication of whether the analysis strategy was “intention to treat” or, if not, description of how non-compliers were treated in the analyses	V	12 to 13 - Sample Size
		For each primary and secondary outcome, a summary of results for each estimation study condition, and the estimated effect size and a confidence interval to indicate the precision	V	25-30 - Tables and Figures
		Inclusion of null and negative findings	V	25-30 - Tables and Figures
Outcomes and estimation	17	Inclusion of results from testing pre-specified causal pathways through which the intervention was intended to operate, if any	V	25-30 - Tables and Figures
Ancilarry analyses	18	Summary of other analyses performed, including subgroup or restricted analyses, indicating which are pre-specified or exploratory	V	25-30 - Tables and Figures
Adverse events	19	Summary of all important adverse events or unintended effects in each study condition (including summary measures, effect size estimates, and confidence intervals)	V	25-30 - Tables and Figures
		**Discussion**		
Interpretation	20	Interpretation of the results, taking into account study hypotheses, sources of potential bias, imprecision of measures, multiplicative analyses, and other limitations or weaknesses of the study	V	15 - Discussion
		Discussion of results taking into account the mechanism by which the intervention was intended to work (causal pathways) or alternative mechanisms or explanations	V	16 - Discussion
		Discussion of the success of and barriers to implementing the intervention, fidelity of implementation	V	16 - Discussion
		Discussion of research, programmatic, or policy implications	V	17 - Discussion
Generalizability	21	Generalizability (external validity) of the trial findings, taking into account the study population, the characteristics of the intervention, length of follow-up, incentives, compliance rates, specific sites/settings involved in the study, and other contextual issues	V	18 - Discussion
Overall Evidence	22	General interpretation of the results in the context of current evidence and current theory	V	19 - Discussion
