# Supplementary figures and images for: Enhancing of Women Functional Status with Metabolic Syndrome by Cardioprotective and Anti-Inflammatory Effects of Combined Aerobic and Resistance Training
Source: PLoS One. 2014 Nov 7;9(11):e110160. doi: 10.1371/journal.pone.0110160 (PMC4224372; doi:10.1371/journal.pone.0110160)

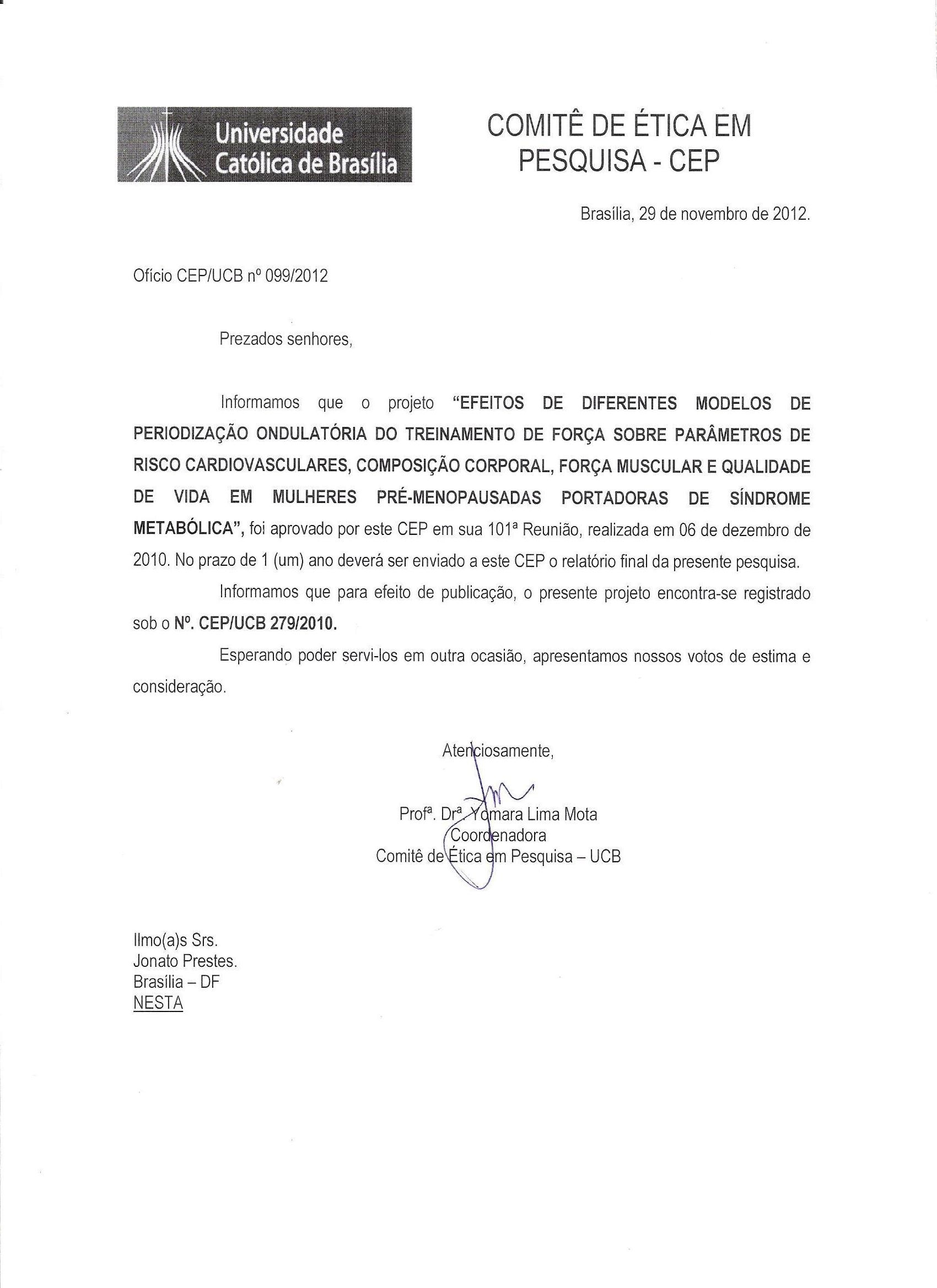

Supplement: Protocol S1 — Copy of the original protocol. (JPG) [file pone.0110160.s002.jpg]
